# Anterior cruciate ligament graft tensioning. Is the maximal sustained one-handed pull technique reproducible?

**DOI:** 10.1186/1756-0500-4-244

**Published:** 2011-07-20

**Authors:** Barry J O'Neill, Fergus J Byrne, Kieran M Hirpara, William F Brennan, Peter E McHugh, William Curtin

**Affiliations:** 1 Department of Orthopaedic & Trauma Surgery, Galway Regional Hospitals, Galway, Ireland; 2 The National Centre for Biomedical Engineering, National University of Ireland, Galway, Ireland

## Abstract

**Background:**

Tensioning of anterior cruciate ligament (ACL) reconstruction grafts affects the clinical outcome of the procedure. As yet, no consensus has been reached regarding the optimum initial tension in an ACL graft. Most surgeons rely on the maximal sustained one-handed pull technique for graft tension. We aim to determine if this technique is reproducible from patient to patient.

**Findings:**

We created a device to simulate ACL reconstruction surgery using Ilizarov components and porcine flexor tendons. Six experienced ACL reconstruction surgeons volunteered to tension porcine grafts using the device to see if they could produce a consistent tension. None of the surgeons involved were able to accurately reproduce graft tension over a series of repeat trials.

**Conclusions:**

We conclude that the maximal sustained one-handed pull technique of ACL graft tensioning is not reproducible from trial to trial. We also conclude that the initial tension placed on an ACL graft varies from surgeon to surgeon.

## Findings

Rupture of the anterior cruciate ligament (ACL) is a common injury in active people. The healing response of ruptured ACL is poor, and without surgical reconstruction the ACL deficient knee limits patient activity and can lead to future degenerative changes [1-4]. In ACL reconstruction surgery it is important to determine how much initial tension should exist in an ACL graft when the knee is unloaded. This tension affects the surgical outcome of the reconstructed knee [5-9]. Proper graft tensioning may be important for restoring normal anteroposterior laxity in ACL reconstruction at the time of graft fixation [10,11].

The optimum amount of force applied to the graft prior to fixation is a matter of considerable debate, with most authors recommending between 20 and 90 N of initial graft tension [9,12-18]. The current clinical practice seems to follow the general guidelines proposed by laboratory based studies, that the average initial graft tension for hamstring tendon grafts used by surgeons is 70 N [11]. The average normal initial tension used by sports medicine trained orthopaedic surgeons is 60 +/-29 N [19]. Although there is some evidence to support isometry [20], it remains a controversial area and many surgeons prefer to tension ACL grafts using manual feedback to determine the amount of tension applied to the graft at fixation. This technique, referred to as the unmeasured initial tension or maximal sustained one-handed pull technique, is currently the most commonly used tensioning protocol and has produced generally good clinical results [9,19,21,22].

### Aims

Cunningham et al published a study in 2002 that demonstrated that graft tensioning is highly variable among sports medicine trained orthopaedic surgeons [19]. To our knowledge, no study has yet assessed the reproducibility of a single surgeon's technique for tensioning ACL grafts. Our aim was to demonstrate that experienced ACL reconstruction surgeons are unable to consistently apply the same amount of tension to an ACL graft using the maximal sustained one handed pull technique, even under controlled laboratory conditions with patient related variables eliminated. We also aimed to demonstrate that the level of tension exerted on an ACL graft at implantation varied from surgeon to surgeon. We hypothesised that even experienced ACL surgeons would be unable to exactly replicate the amount of tension applied to an ACL graft on multiple occasions, and that each surgeon would apply a different mean tension to the graft when compared with the other surgeons.

## Materials and methods

**Figure 1:**
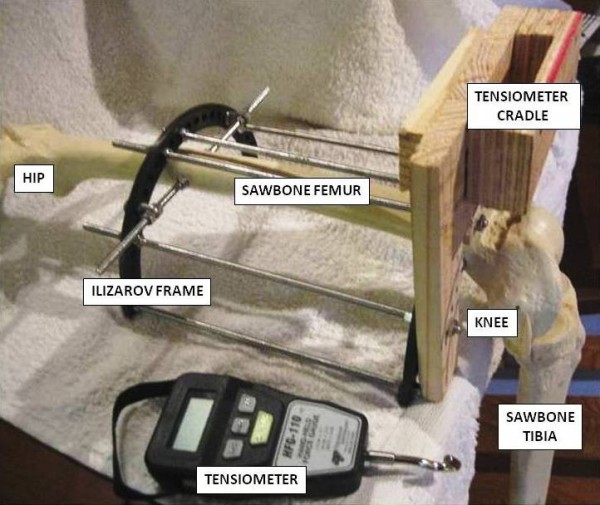
**Portable testing frame with sawbone femur and tibia**.

A specially designed frame was constructed using used and sterilised Ilizarov components. This frame consisted of a semi-tubular structure made of half-rings and rods, with attachments dropped from the rings to accommodate a femur (Figure 1). The femur was a cortical shell sawbone femur attached to a cortical shell sawbone tibia by nylon cord attachments to represent the posterior cruciate and collateral ligaments (Type 1146, Sawbones Europe AB, Malmo, Sweden). The sawbones are entirely made of rigid polyurethane foam that simulates cancellous bone. The mechanical properties of the sawbones were found to be in the range of normal bones [23]. The femur was encased within the frame, but the tibia was free. When the frame was positioned on the edge of a table, the tibia could be manipulated through a range of flexion and extension. This represented the position of the leg when undergoing ACL reconstruction in the supine position.

A guide-wire was passed through the tibia at 15 degrees from the sagittal plane and 45 degrees from the axial plane as described by Asagumo et al for placement of the anteromedial bundle of an ACL graft [24]. The guide-wire was passed through the centre of the tibial ACL footprint as described by Sapega et al [25], and then advanced directly through the femur with the knee flexed to 100 degrees. A cannulated 8 mm drill was then passed over the guide-wire to form the tibial and femoral tunnels.

**Figure 2:**
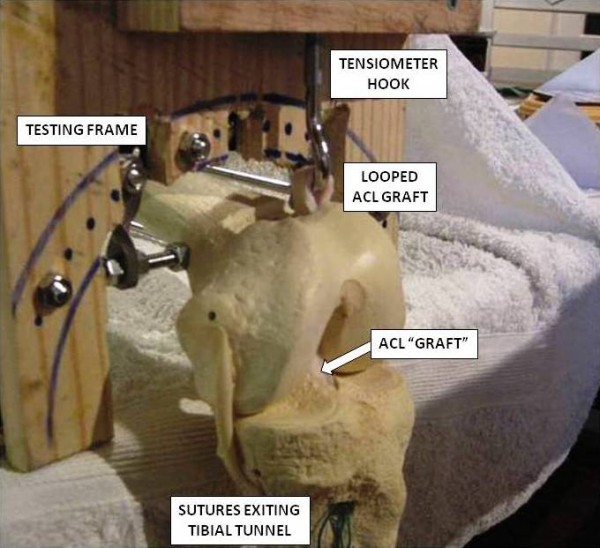
**ACL 'graft' looped around hook of tensiometer**.

**Figure 3:**
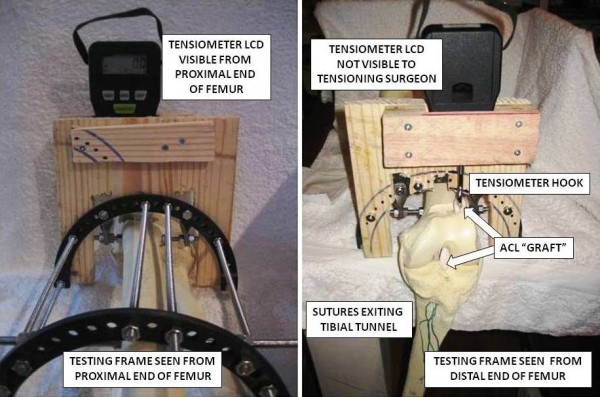
**Tensiometer LCD as seen from proximal and distal**.

A hand held strain tensiometer (HFG-110 Hand-Held Force Gauge, Transducer Techniques, Temecula, CA, USA) was attached to the testing frame directly above the exit point of the femoral tunnel. A stainless steel hook projected down from the tensiometer, in order that an ACL 'graft' could be looped around the hook, to represent a double-bundle hamstring graft (Figure 2). The Liquid Crystal Display (LCD) showing the tension recorded through the graft-tensiometer complex was positioned such that it was visible from the proximal end of the 'femur', but not from the knee end (Figure 3). Any subject tensioning a graft complex was therefore blinded to how much tension they were imparting upon the graft. The tension recorded could be documented by an independent observer standing at the proximal end of the frame.

The ACL graft consisted of a double-bundle porcine flexor digitorum tendon [26] harvested from 85-100 Kg pigs ranging in age from 6 to 8 months [27]. Pig trotters were sourced from a local abattoir and each tendon was harvested within 24 hours of slaughter. A whip stitch was placed at both ends of each tendon using a No. 5 Ethibond suture (Ethibond, Somerville, NJ, USA). Each tendon was bathed in a 0.9% saline solution at room temperature for seven to eight minutes, then placed in sealed plastic bags and stored at -25 degrees centigrade. Tendons were thawed for 12 hours in 0.9% saline solution at room temperature. A 20-lb (9 kg) load was applied to each tendon for 5 minutes to reduce creep and slippage between the tendon and suture prior to testing.

Tendons were selected that were all 120 mm in length, and when doubled could pass through an 8 mm diameter stainless steel tube. The ACL graft was then passed through the tibial and femoral tunnels (8 mm diameter) and looped over the tensiometer hook, to represent a hamstring double bundle graft. Six consultant orthopaedic & trauma surgeons agreed to participate in the study. These six surgeons had been registered specialists in orthopaedic surgery for a mean 11.5 years (Range 4-30 years). They performed on average 51 ACL reconstructions each year (Range 30-150). The testing apparatus was taken to each surgeon, and was then secured to a desk or table, with the tibia hanging freely over the edge of the table. Each surgeon was then asked to tension the ACL graft exactly as he would when performing an ACL reconstruction procedure. No other instruction was given, and each surgeon was free to move the 'leg' and tension the graft exactly as he wished. Each surgeon was blinded to the LCD. When he was satisfied with the tension within the graft, the tension on the LCD was documented. The participating surgeon was blinded to each result. This process was repeated 5 times for each surgeon at 60 second intervals.

Prior to commencing this study the guidelines of the institute's Research Ethics Committee were reviewed and advice procured from the research office. The study involved professional voluntary participants. No live human or animal subjects were involved. The study did not directly affect or influence clinical care of human subjects. After consideration the authors were advised that application for Research Ethics Committee approval was not necessary under the institute's published research guidelines.

## Results

**Figure 4:**
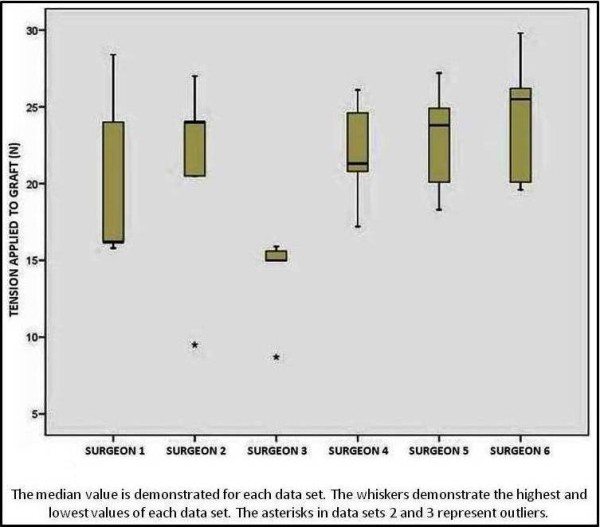
**Range of outcomes for tensioning experiments**.

All of the six participating surgeons opted to use the maximal sustained one handed pull technique. Each confirmed that this was their preferred method of tensioning an ACL graft. The results of the tensioning experiments can be seen in Table 1. Surgeon number 3 consistently applied a very similar amount of tension on all but one occasion. The other five surgeons applied a very variable amount of tension through their five attempts (Figure 4).

**Table 1:** Tension applied by consultant surgeons (N)

TENSION APPLIED BY CONSULTANT SURGEONS (N)						
	**TENSION 1**	**TENSION 2**	**TENSION 3**	**TENSION 4**	**TENSION 5**	**MEAN**

SURGEON 1	24.0	28.4	16.2	18.5	15.8	20.58

SURGEON 2	9.5	20.5	24.0	24.0	27.0	21

SURGEON 3	15.9	15.0	8.7	15.6	15.0	14.04

SURGEON 4	21.3	24.6	20.8	17.2	26.1	22

SURGEON 5	24.9	20.1	27.2	18.3	23.8	22.86

SURGEON 5	26.2	29.8	25.5	19.6	20.1	24.24

Coefficients of variation (CV) and Standard Errors of Measurement (SEM) were calculated for each participating surgeon. A CV of less than 10% was considered to represent reliable reproducibility, CV of greater than 10% representing poor reproducibility. The CV and SEM for each surgeon can be seen in Table 2.

**Table 2:** Co-efficients of variations and standard errors of measurement for all six surgeons

COEFFICIENTS OF VARIATION & STANDARD ERRORS OF MEASUREMENT FOR ALL 6 SURGEONS						
**PARAMETER**	**SURGEON 1**	**SURGEON 2**	**SURGEON 3**	**SURGEON 4**	**SURGEON 5**	**SURGEON 6**

MEAN (N)	20.580	21.000	14.040	22.000	22.860	24.240

SD	5.459	6.828	9.010	3.484	3.616	4.330

CV	27%	33%	21%	16%	16%	18%

SEM	2.44 N	3.05 N	1.34 N	1.55 N	1.61 N	1.93 N

90%CI	15.38-25.78	14.49-27.51	11.17-16.91	18.68-25.32	19.41-26.31	20.11-28.37

95% CI	13.80-27.36	12.52-29.48	10.30-17.78	17.68-26.33	18.37-27.35	18.86-29.62

99% CI	9.34-31.82	6.94-35.06	7.84-20.24	14.83-29.17	15.42-30.31	15.32-33.156

MINIMUM (N)	15.8	9.5	8.7	17.2	18.3	19.6

MEDIAN (N)	18.5	24	15	21.3	23.8	25.5

MAXIMUM (N)	28.4	27	15.9	26.1	27.2	29.8

## Discussion

The outcome of ACL reconstruction surgery is influenced by many variables. Graft choice [28,29], tunnel placement [30,31], graft tensioning [5-11] and graft fixation [32,33] have all been shown to influence the short and long-term outcome of ACL reconstruction surgery. The purpose of this study was to assess the reproducibility of the maximal sustained one handed pull technique only. We are not suggesting that tensioning is the only important aspect of ACL reconstruction surgery, and we are not suggesting that it is any more important than any other factor.

It has been demonstrated that small changes in the initial tension within an ACL graft cause significant differences in stability [34,35] and range of motion [34]. An under-tensioned graft will not restore knee stability [34]. An over-tensioned graft will pull the femur anteriorly on the tibia and restrict the range of motion within the knee [34]. This places the graft at increased risk of damage during normal physiological loading [20,36]. The optimum initial graft tension has yet to reach consensus. The mean initial graft tension employed by orthopaedic surgeons is 70 N (Newtons) for hamstring grafts and 47 N for B-PT-B grafts, with a range of 20 N to 80 N [11]. This wide range of values is in part due to the different protocols and laboratory conditions under which tensioning has been tested [8,10,11,20,34-39].

In a clinical situation there are a number of factors that will influence the initial tension placed on an ACL graft. The maximal sustained one handed pull technique relies on feedback from the graft to the surgeon, and the surgeon's interpretation of the feedback. This will in turn be influenced by the size of the patient, by the size and length of the graft material, by the size and length of the bone tunnels, and therefore the surface contact area between the graft and the bone tunnels, by the quality of the bone and the graft material being used, and by the accuracy of tunnel placement. Some of these variables are dependent upon the skill of the surgeon, but many are patient factors that cannot be controlled by the operating surgeon.

This study allowed us to eliminate some of the 'patient' factors inherent in ACL reconstruction surgery. The testing jig was identical for each experiment. Each surgeon used the same sawbone and porcine tendon for their series of tests. This eliminated any difference between tests in the 'bone' quality, the tunnel alignment or placement, the length, circumference, and quality of the tendon, and the interaction between the tendon and the sawbone. Each sawbone used was prepared in exactly the same way, and the tunnels were all drilled in an identical fashion. Each tendon was the same length and circumference and was prepared in an identical fashion. There may have been minor differences in the quality of the sawbones and tendons used for each series of tests, but the main focus of the study was to determine whether or not a single surgeon could accurately reproduce a set tension within an ACL reconstruction graft under controlled conditions.

The actual tensioning figures produced by each surgeon are of secondary importance, as we have already stated that the optimum initial tension that should be imparted upon the graft has not reached consensus, and that individual surgeons vary greatly in their desired initial tension. None of the surgeons were able to consistently reproduce tension in the graft under these controlled conditions. Surgeon number 3 had a very consistent series of test on all but one occasion. The other five surgeons have markedly variable results. These results were obtained under controlled experimental conditions, and we would conclude that under clinical conditions with multiple other variables introduced into the tensioning procedure, the results would be even less consistent. Even surgeon number three who had markedly similar results on all but one occasion, was unable to explain why he had one result which was markedly different from the others. The feedback that he got back from the graft was the same on all five occasions.

We must acknowledge certain limitations within this study. Only six orthopaedic surgeons were included within the study. This is a small number and ideally would have been larger. We were unable to contact/include further surgeons with the necessary clinical experience for inclusion during the timescale of the experiments.

We chose to use sawbones over cadaveric bones. We accept that sawbones do not simulate the mechanical properties of cadaveric bone [40], but the use of sawbone in orthopaedic research is common and has been validated [40,41]. It was felt that a new femur and tendon should be used for each set of experiments, to prevent any difference in graft-tunnel friction that may affect the results. It was felt that in order to limit the number of variables inherent in each trial, that sawbones would provide a more uniform material for each experiment, as each was manufactured in exactly the same way and the tunnels drilled in exactly the same fashion each time. The different densities of cadaveric bone from two or more different specimens, related to the age and activity level of the donors, would have introduced an unnecessary variable [42,43]. The testing apparatus was designed to be portable, in order that it could be used by six surgeons at five different institutions over a period of twelve weeks. It was felt that bringing a thawed cadaveric bone to five different healthcare institutions represented an un-necessary infection risk [44,45].

We also accept that the hand-held tensiometer and testing frame set-up would not directly measure the tension within the graft, as the frictional effect of the graft-tunnel interface is an unknown. The purpose of the experiment however is to assess the reproducibility of the maximal sustained one handed pull technique, and so the absolute tension within the graft is being indirectly measured if the other variables, including the frictional effect of the graft-tunnel interface remain constant throughout testing.

This study did not intend to assess the effect of graft fixation on the tensioning of the graft, or to assess the long term outcomes of graft tensioning on knee range of motion or joint laxity. These aspects of ACL reconstruction surgery are dealt with in other published trials, some of which have been referenced.

## Conclusions

These results demonstrate that even under closely controlled conditions, experienced ACL reconstruction surgeons are not able to consistently tension an ACL graft using the maximum sustained one-handed pull technique. This was the technique favoured by all six surgeons in this study, and is the technique favoured by the majority of ACL surgeons. There are commercially available devices that assist in graft tensioning, but to our knowledge all of these devices rely on the operating surgeon's interpretation of feedback from the graft. To our knowledge, no device yet exists that sets the tension within an ACL graft to a predetermined level.

## Competing interests

The authors declare that they have no competing interests.

## Authors' contributions

BJON developed the idea for testing of tensioning, designed and built the testing frame, conducted the experiments tensioning experiments with consultant surgeons, and drafted the manuscript. FJB assisted with drafting and editing of the manuscript. KMH assisted with statistical analysis and editing of the manuscript. WFB assisted with the conception of the study and the design of the testing frame. PEMcH assisted with the concept and design of the study and oversaw the technical aspects of the testing. WC assisted with the concept and design of the study, helped design the testing frame, participated in the testing protocol, assisted with editing of the manuscript, and supervised the entire study. All authors read and approved the final manuscript.
